# Post-Heat Flexural Properties of Siloxane-Modified Epoxy/Phenolic Composites Reinforced by Glass Fiber

**DOI:** 10.3390/polym16050708

**Published:** 2024-03-05

**Authors:** Yundong Ji, Xinchen Zhang, Changzeng Wang, Shuxin Li, Dongfeng Cao

**Affiliations:** 1 School of Materials Science and Engineering, Wuhan University of Technology, Wuhan 430070, China; jiyundong@whut.edu.cn (Y.J.); 18084008671@163.com (X.Z.); czwang@whut.edu.cn (C.W.); 2 State Key Laboratory of Advanced Technology for Materials Synthesis and Processing, Wuhan University of Technology, Wuhan 430070, China; lishuxin@whut.edu.cn; 3 Hubei Key Laboratory of Theory and Application of Advanced Materials Mechanics, Wuhan University of Technology, Wuhan 430070, China; 4 Foshan Xianhu Laboratory of the Advanced Energy Science and Technology Guangdong Laboratory, Foshan 528000, China; 5 Institute of Advanced Materials and Manufacturing Technology, Wuhan University of Technology, Wuhan 430070, China

**Keywords:** phenolic sin, polysiloxane-modified epoxy, post-heat mechanical strength, fire resistance, glass fiber composite

## Abstract

The post-heat mechanical property is one of the important indices for the fire-resistance evaluation of fiber-reinforced polymers. At present, the primary approach to improving the post-heat mechanical property of a material involves incorporating inorganic fillers; yet, the enhancement is limited, and is accompanied by a reduction in room-temperature performance and processability. This study prepares glass-fiber-reinforced composites with elevated mechanical properties after heat through utilizing two variants of epoxy resins modified with polysiloxane, phenolic resin, kaolin, and graphite. In comparison to the phenolic samples, the phenylpropylsiloxane-modified epoxy resulted in a 115% rise in post-heat flexural strength and a 70% increase in the room-temperature flexural strength of phenolic composites. On the other hand, dimethylsiloxane-modified epoxy leads to a 117% improvement in post-heat flexural strength but a 44% decrease in the room-temperature flexural strength of phenolic composites. Macroscopic/microscopic morphologies and a residual structure model of the composites after heat reveal that, during high temperature exposure, the pyrolysis products of polysiloxane promote interactions between carbon elements and fillers, thus preserving more residues and improving the dimensional stability as well as the density of materials. Consequently, a notable enhancement is observed in both the post-heat flexural strength and the mass of carbon residue after the incorporation of polysiloxane and fillers into the materials. The pyrolysis products of polysiloxane-modified epoxy play a vital role in enhancing the post-heat flexural strength by promoting carbon retention, carbon fixation, and interactions with fillers, offering novel pathways for the development of advanced composites with superior fire-resistance properties.

## 1. Introduction

For the past few years, the extensive utilization of glass-reinforced polymer composites has been witnessed across diverse sectors such as shipping, aerospace, and construction. These materials are favored for their moderate cost, light weight, high strength, excellent corrosion resistance, and excellent mechanical properties [1,2,3,4]. Nevertheless, these composites exhibit combustibility and a relatively high rate of smoke generation [4]. At elevated temperatures, the thermal decomposition and vigorous combustion of the matrix induce dimensional alterations and the deterioration of the mechanical properties of composites. In the case of combustion, composites can hardly maintain their original structure, bringing significant safety risks for evacuees and fire rescue crews. Therefore, the mechanical properties of composites after fire are extremely worthy of assessment and enhancement.

In 2018, the Marine Technology and Shipbuilding Commission (F25) of the United States issued an updated standard specification (ASTM F3059-18 [5]) for the fiber-reinforced polymer (FRP) gratings used in marine architecture and shipbuilding. On the one hand, the standard outlined the precise test temperature and method of the post-fire mechanical properties. On the other hand, it incorporated the post-fire mechanical properties into the fire-resistance index of composites, indicating that the fire-resistance evaluation of composites is not only focused on the conventional fire-resistance indices such as oxygen index and flame spread rate but also involves the post-fire mechanical properties. It is crucial that these indices determine the suitability of composites for high-temperature applications.

Several researchers have fabricated glass fiber composites based on epoxy and polyester; they have also fabricated phenolic and silica fiber composites based on phenolic and have made carbon fiber composites based on phenolic as well as bentonite/phenolic. In addition, the thermomechanical response of the composites mentioned above at and after high temperatures was also discussed.

Mouritz et al. [1] employed a cone calorimeter to heat glass-fiber-reinforced polyester, epoxy, and phenolic composites. After four minutes of ablation with a heat flux of 50 kW/m^2^, the flexural strengths of chopped glass fiber/polyester composites, chopped glass fiber/phenolic composites, and chopped glass fiber/epoxy composites were approximately 37.5 MPa, 20 MPa, and 5 MPa, respectively. Additionally, the flexural performances of glass-fiber-reinforced polyester, phenolic, and vinyl ester were investigated [2] after 30 min of exposure to a heat flux of 50 kW/m^2^. The results reveal that, under a long-term heat condition, phenolic composites have more significant superiority over polyester and vinyl ester composites.

Gardiner et al. [6] investigated the flexural performance of glass-reinforced polyester (GRP) panels after fire, where the panels were burnt by a 10 min kerosene flame. In cases where the burnt side was subjected to bending-induced tension and compression, the post-fire flexural strengths of the composites were about 35 Mpa and 15 Mpa, respectively. A model considering combustion panels as double-layer materials was also developed to assess the structural deterioration of GRP ship construction after fire.

Gibson et al. [7] conducted an experiment about the post-fire mechanical properties of polyester/glass and phenolic/glass floor gratings, which was subjected to a heat flux of 37.5 kW/m^2^ brought by a propane burner for 16 min. The post-fire flexural strengths of these two floor gratings were 0 MPa and 34 MPa, respectively. In comparison to polyester/glass floor gratings, the research results indicate that phenolic/glass floor gratings show a longer ignition time, lower flame and smoke emission, and higher post-fire flexural strength. Additionally, the study confirms that a structural strength of 100 MPa after fire is necessary for bending resistance.

Shi et al. [8] examined the effects of different temperatures on the compressive performance of silica-fiber-reinforced phenolic composites within a circular furnace. The composites displayed a compressive strength of 100 MPa at 800 °C. Furthermore, the researchers established a thermomechanical model to forecast the compressive strength and the failure time of the composites. The calculated and tested outcomes showed good consistency.

Asaro et al. [9] investigated the mechanical properties of carbon-fiber-reinforced phenolic composites and carbon-fiber-reinforced bentonite/phenolic composites after fire. After 10 min of combustion in a cone calorimeter at 50 kW/m^2^, it was found that the flexural performance of composites decreased by 89% and 96% in comparison with samples at room temperature. This outcome is due to the pyrolysis of resin and the delamination between matrix and fiber.

According to the literature described above, the flexural performance of glass-fiber-reinforced phenolic composites has been found to be lower than that of glass-fiber-reinforced polyester composites after short-term heat. However, the former exhibited superior flexural performance compared to the latter after prolonged heat. In comparison to composites based on epoxy, polyester, and other polymers [10,11,12], phenolic composites demonstrate superior post-heat strength. Even so, further enhancement of the post-heat strength of composites is necessary, alongside improving their poor room-temperature strength. The post-heat mechanical properties of composites are remarkably influenced by the oxidative pyrolysis of the phenolic at high temperature [13,14,15], as well as the content, composition, and structure of the pyrolysis products. For example, a reduction in char yield, an increase in macro- and micropores, the dimensional expansion of materials, and the weak connections between fibers and matrix all contribute to the deterioration of mechanical properties for polymer-based composites.

ZrB_2_ [16], amorphous silica balls [17], kaolin [18], and microcrystalline muscovite [19] along with other inorganic solid fillers, are capable of sintering with char to some extent, thereby enhancing the carbon content, the densification of residual structures, and the residual mechanical properties [16,17,18,19] after burning. However, this process is accompanied by a reduction in the manufacturability and mechanical performance of composite materials at room temperature [19,20,21]. In addition, the phenolic resin can be toughened by incorporating epoxy resin [22,23] and rubber [24,25,26], while this adjustment may come at the expense of its high-temperature performance. Therefore, eliminating the contradiction between high-temperature and room-temperature performance becomes the objective of matrix modification.

To tackle this contradiction, the research group has deliberated the exceptional thermal stability [27,28,29,30] and mechanical properties [31] of siloxane-modified epoxy, as well as the sintering and fire resistance of kaolin [18,32]. Subsequently, a proposal has been formulated to introduce the fire-resistant elements (Si) through intrinsic siloxane-modified epoxy [32,33,34,35,36] and inorganic fillers containing kaolin [32]. This approach aims to simultaneously enhance the post-heat mechanical properties and fire resistance of materials, as well as the mechanical performance of materials at room temperature. Especially at elevated temperature, residues from siloxane-modified epoxy can sinter with fillers such as kaolin [32], thereby achieving the objectives of carbon preservation, structural densification, and increased mechanical properties of the composites after heat.

Consequently, this article performs a further study on the previous research of hydroxyl-terminated polydimethylsiloxane-modified epoxy/phenolic composites [32] by conducting additional research on phenylpropylsiloxane-modified epoxy/phenolic composites. To introduce silicon elements, the phenolic resin is blended with these two kinds of siloxane-modified epoxies, which possess intrinsic flame retardancy. A series of inorganic fillers such as kaolin and graphite are also added to fabricate two types of glass-fiber-reinforced polymers. In accordance with ASTM F3059-18 [5], the time–temperature curve and testing conditions during actual fires are approximately simulated. Moreover, the flexural mechanical properties of composites at room temperature and after 1 h heat, along with the macroscopic and microscopic morphology after heat, are compared and analyzed. Ultimately, the thermal decomposition processes and post-heat residual structure model for the composites are presented. The innovation and novelty of this article lie in the enhancement of the room-temperature and post-heat mechanical properties of composites, as well as the interaction between siloxane and inorganic fillers in composites under high temperature. It has been discovered that the incorporation of phenylpropylsiloxane-modified epoxy can enhance the mechanical properties of phenolic composites at room temperature. Furthermore, this article indicates that the two types of siloxane-modified epoxy can serve as organic precursors of C/Si/O hybrid pyrolysis products into the composites. These uniformly distributed “in situ” pyrolysis products play an important role in carbon preservation and fixation, and under high-temperature condition, they undergo co-sintering with inorganic fillers, thereby significantly enhancing the post-heat mechanical properties. This undoubtedly offers a fresh perspective for the design of advanced flame-retardant composites with superior mechanical properties at both room temperature and high temperatures.

## 2. Experiment

### 2.1. Experimental Materials

Bisphenol A type epoxy resin (E51): industrially pure, with an epoxy value of 0.51~0.54 mol/100 g, produced by Baling Petrochemical Branch of Sinopec Group, Yueyang, China. Barium-catalyzed phenolic resin (PF-7201): industrially pure, produced by Hubei Lifa Co., Ltd., Wuhan, China. Hydroxyl-terminated linear polydimethylsiloxane (SH-203-F): industrially pure, with a hydroxyl content of 6–8 wt% and a viscosity (25 °C) of 19.2–38.4 mPa·s, produced by Hubei Longsheng Sihai New Materials Co., Ltd., Xiangyang, China. Hydroxyl-terminated polyphenylpropylsiloxane (Z-6018): sheet-like solid, produced by Dow Corning Company, Midland, TX, USA. Oxalic acid: analytically pure, produced by China National Pharmaceutical Group Chemical Reagent Co., Ltd., Shanghai, China. Bismuth isooctanoate: analytically pure, produced by China National Pharmaceutical Group Chemical Reagent Co., Ltd., Shanghai, China. Dibutyltin dilaurate (DBTDL): chemically pure, produced by China National Pharmaceutical Group Chemical Reagent Co., Ltd., Shanghai, China. Kaolin: chemically pure, produced by China National Pharmaceutical Group Chemical Reagent Co., Ltd., Shanghai, China. Graphite powder: 99.95% metal basis, 80–120 mesh, produced by Shanghai McLean Biochemical Technology Co., Ltd., Shanghai, China. Alkali-free unidirectional glass fiber bundle (ECR469L-2400): with a linear density of 2400 g/km, produced by Chongqing International Composites Co., Ltd., Chongqing, China.

### 2.2. Material Preparation

#### 2.2.1. Polyphenylpropylsiloxane-Modified Epoxy Resin (EPh)

**Figure 1:**
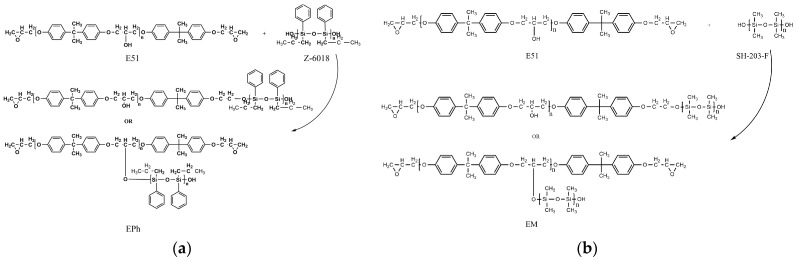
The chemical reaction of Eph (**a**) and EM (**b**).

Following the prior research group’s work on EPh synthesis [33] and further exploration of the synthesis process, a 3:7 ratio of Z-6018/epoxy resin was selected. The cured resin displays uniform transparency, good heat resistance, and excellent mechanical properties. The preheated epoxy resin and Z-6018 were combined in a 1000 mL three-necked flask containing a thermometer, a condenser tube, and a mechanical mixer. Next, DBTDL was gradually introduced into the mixture, and the mechanical mixer was increased to 200–300 r/min for a two-hour reaction; the temperature was evaluated for an additional hour of reaction. Lastly, the mixture was subjected to vacuum heating until a constant weight was achieved to obtain EPh. Figure 1a shows the chemical reaction of EPh.

#### 2.2.2. Polydimethylsiloxane-Modified Epoxy Resin (EM)

The previous research about EM synthesis [34] has found that a 3:7 ratio of SH-203-F/epoxy resin, with high Si content, produces an opalescent emulsion with uniform color. The preheated epoxy resin and weighed SH-203-F were added to a 1000 mL three-neck flask equipped with a thermometer, a condenser tube, and a mechanical mixer. The oil bath was heated to 80 °C, and the mechanical mixer was set to 200~300 r/min. After 30 min of reaction, a specific amount of oxalic acid was slowly added, and the flask was vacuumed for 30 min. The temperature of the oil bath was then adjusted until the solution reached 95 °C. After stopping the vacuum, a specific amount of bismuth isooctanoate was added. The vacuum was then maintained, and the temperature was raised to 120 °C. After two hours, the synthesis reaction was complete, resulting in the production of EM. Figure 1b shows the chemical reaction of EM.

#### 2.2.3. Preparation of Resin Blends

According to the previous study, which was conducted by the research group who published [35], the resin was weighed in mass ratios of PF-7201 (10, 60 g), E51:PF-7201 (3:7, 18 g:42 g), EPh:PF-7201 (3:7, 18 g:42 g) and EM:PF-7201 (3:7, 18 g:42 g), and then thoroughly mixed through mechanical mixing. Subsequently, the resin underwent vacuum extraction and defoaming treatment in an oven, labeled as PF, E51/PF, EPh/PF, and EM/PF, respectively. Owing to the fact that the phenolic hydroxyl hydrogen in the phenolic resin is a highly reactive hydrogen capable of undergoing ring-opening polymerization with the epoxy group in the epoxy resin under specific temperature conditions [37], the resin blends can be crosslinked and cured without any other curing agents. Once the resin was ready for use, it was poured into the mold and cured at 120 °C for 1 h, 150 °C for 3 h, and 170 °C for 2 h, respectively, all under a pressure of 10 MPa.

#### 2.2.4. Preparation of Glass-Fiber-Reinforced Composites

**Figure 2:**
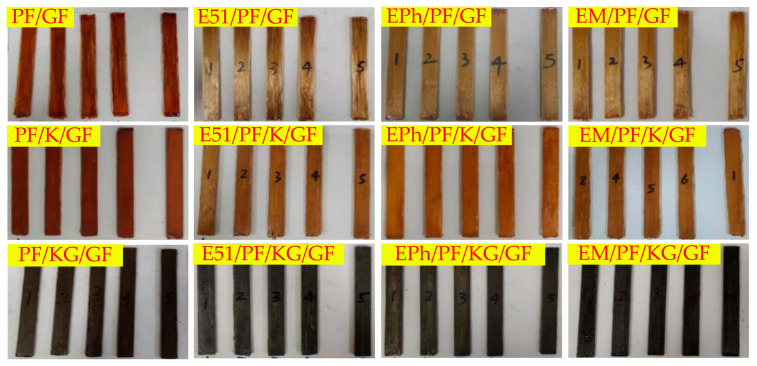
Physical appearances of composites.

Fillers including kaolin and kaolin/graphite (inorganic fillers (about 30 g) comprising 50 wt% of the resin, with kaolin/graphite (24 g/6 g) mixed in a 4:1 mass ratio for 10 min using a mechanical mixer), were gradually introduced into resin systems, such as PF, E51/PF, EPh/PF, EM/PF, etc., multiple times. A duration of 15 min was needed each time for mechanical mixing, then an appropriate amount of filler was incorporated, culminating in vacuuming and defoaming the resin-filler blends. We began by cutting a unidirectional fiberglass fabric (about 270 g) of the specified dimension and placed it in the mold in one orientation. Next, the necessary amount of resin or resin-filler blend was applied using a scraper to evenly distribute it along the direction of the fibers and fully saturate them. This procedure was repeated for each layer of fibers in a comparable fashion. Lastly, the mold was placed in a flat vulcanization machine for hot pressing and curing at a pressure of 10 MPa. The temperature conditions were set at 120 °C for 1 h, 150 °C for 3 h, and 170 °C for 2 h, individually. After demolding, the composites were processed into standard-dimension specimens using the RS-6590 engraving machine from Lockheed Electric. Graphite, kaolin, and glass fiber are represented as G, K, and GF, respectively. There are three composite systems labelled with N-Composite (No filler), K-Composite (contain kaolin), and KG-Composite (contain kaolin/graphite), respectively. Table 1 presents the abbreviations of the composites and the mass of components used in this study, while Figure 2 shows their physical appearance. To minimize the influence of sample dimension and resin content on the post-heat residual strength of composites, the width, height, and resin content of the samples were precisely regulated. The pre-heat dimensions of the composites were specified as 15 mm in width (with a tolerance of ±0.5 mm) and 3 mm in height (with a tolerance of ±0.2 mm). The resin content in the composites was held at 18 wt% (with a tolerance of ±5 wt%).

### 2.3. The Heat Treatment of Flexural Specimens

**Figure 3:**
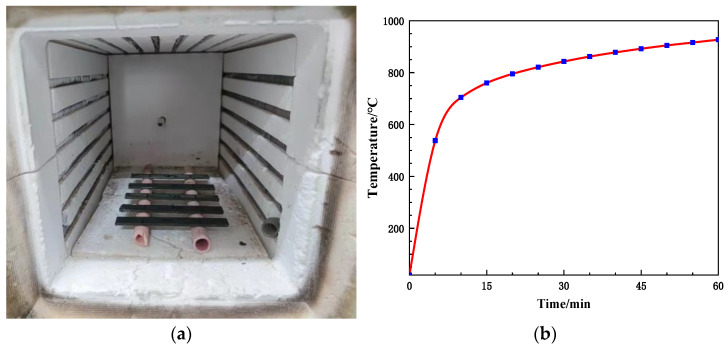
Composites in the furnace (**a**) and the time–temperature curve of ASTM 3059-18 [5] (**b**).

Before heat treatment, the mass and dimensions of the composites were recorded, consisting of 5 test specimens together. According to the ASTM 3059-18 [5], the samples were positioned on a 60 mm span hollow frame within the atmosphere furnace of the Shanghai DROIDE SA2-6-12TP type, imitating a hollow environment. The samples within the furnace are illustrated in Figure 3a. Following the time–temperature curve depicted in Figure 3b, the samples were heated from room temperature to 927 °C. Upon completion of the heat treatment, a steel clamp was used to extract the samples; we allowed them to cool in the air until they returned to room temperature. Afterwards, the mass and dimensions of the samples were measured, and the gauge length was marked with a marker pen. Lastly, it was placed in a dry plastic bag for mechanical property testing.

### 2.4. Characterization Methods

#### 2.4.1. Flexural Property Testing

**Figure 4:**
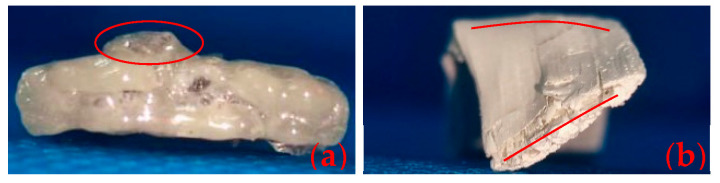
Volcanic pores on the surface (**a**) and distortion of the structure (**b**).

**Figure 5:**
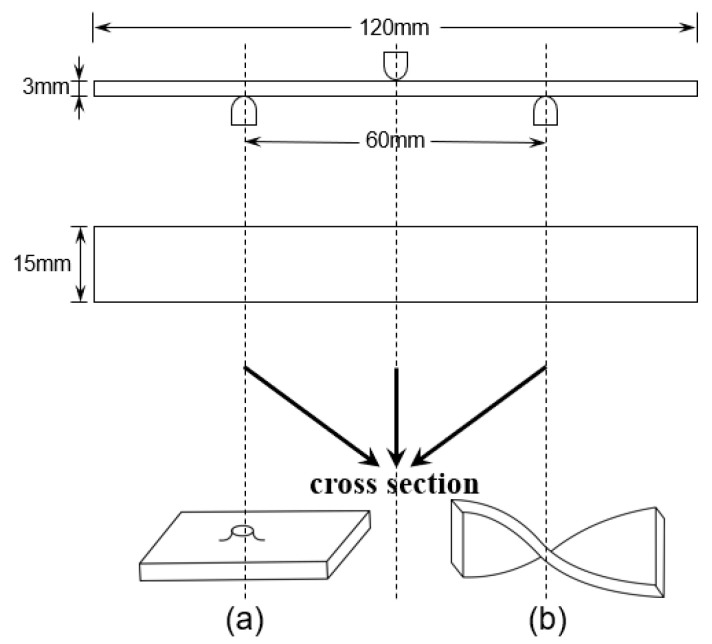
Volcanic pores (**a**) and distortion (**b**) at the position of three-point loading.

The flexural strength of samples was determined at room-temperature and after heat treatment, respectively, to characterize their mechanical properties of various temperatures. It is crucial to concurrently consider the dimension and maximum load when comparing the post-heat flexural strength of the specimens. The nearly identical dimensions of specimens at room temperature leads to a direct correlation between their flexural strength and maximum load. Nevertheless, the alterations in the dimension of specimens after the heat treatment can notably impact their strength values. For instance, specimens with larger maximum load may display decreased strength because of the material expansion, suggesting that the influence of maximum load on strength may be less pronounced than that of dimensional alterations on strength. The testing standard follows EN ISO 14125-1998 [38], with a sample dimension of 120 mm × 15 mm × 3 mm and a 60 mm span. The maximum load and flexural strength were calculated and averaged from 5 specimens. Following the heat treatment, numerous volcanic pores were observed on the surfaces of PF/GF, E51/PF/GF, and other samples, as shown in Figure 4a. Noticeable distortion was observed in the E51/PF/GF, E51/PF/K/GF, EM/PF/K/GF, and E51/PF/KG/GF samples, as depicted in Figure 4b. The dimensions of these samples, presented with volcanic pores and distortion, as measured by a vernier caliper, are obviously enlarged. To reduce the effects, the width and thickness of these samples are taken as the average value of 6 arbitrary positions within the span, while the width and thickness of other samples are the average value of 3 arbitrary positions within the span. Furthermore, some samples may display defects, such as volcanic pores (Figure 5a) and distortion (Figure 5b), at the position of three-point loading, necessitating their exclusion. Testing was conducted by Instron 5967 microcomputer-controlled electronic universal testing machine (Boston, MA, USA). The formula of three-point flexural strength is presented in Equation (1).

(1) $$\sigma =\frac{3FL}{2bh^{2}}$$

Here, σ is flexural strength, F is the maximum load, L is the span, b is the width, and h is the thickness.

#### 2.4.2. The Char Yield

The char yield, to a certain extent, can determine the heat resistance of the samples when exposed to high temperature. Before subjecting the samples to the heat treatment, the mass of resin, inorganic fillers, and glass fiber utilized were measured and documented. Following heat treatment, the mass of the samples were measured and recorded. The pyrolysis of glass fibers and kaolin utilized in this study was negligible, whereas resin and graphite exhibited certain degree of pyrolysis. This article defines the following: the mass ratio of samples before and after heat treatment when removing fibers is the mixture(resin-filler) char yield(R_0_) (the calculation formula is shown in Equation (2)); the mass ratio of samples before and after heat treatment, specifically when eliminating fibers and fillers (consider only the resin) is the resin char yield. The calculation formulas of R_1_(the resin char yield of N-Composite), R_2_(the resin char yield of K-Composite) and R_3_(the resin char yield of KG-Composite) are presented in Equations (3)–(5), respectively.

(2) $$R_{0}=\frac{m_{A}-m_{GF}}{m_{B}-m_{GF}}$$

(3) $$R_{1}=\frac{m_{A}-m_{GF}}{m_{B}-m_{GF}}$$

(4) $$R_{2}=\frac{m_{A}-m_{GF}-m_{K}}{\left(m_{B}-m_{GF}\right)\times 2÷3}$$

(5) $$R_{3}=\frac{m_{A}-m_{GF}-m_{K}-m_{G}\times 11.25\%}{\left(m_{B}-m_{GF}\right)\times 2÷3}$$

In Equations (2)–(5), m_B_ is the mass of samples before heat treatment, m_A_ is the mass of samples after heat treatment, m_GF_ is the mass of fiber, m_K_ is the mass of kaolin, and m_G_ is the mass of graphite. Upon heat treatment according to Figure 3b, glass fiber and kaolin exhibit almost 0% mass loss. In addition, disregarding the reaction between graphite and kaolin, the char yield of graphite in the graphite/kaolin blend is 11.25%.

#### 2.4.3. Morphology

Upon heat treatment, the macroscopic morphology of samples was recorded with a high-definition camera, and their microscopic morphologies were examined by ZEISS Gemini300 scanning electron microscope (SEM, Oberkochen, Germany). Before the scanning electron microscopy testing, the cross-sections of samples were polished and cleaned with a KQ-50DE CNC ultrasonic cleaner for 30 min, and then dried at 80 °C for 1 h.

## 3. Results and Discussion

### 3.1. Flexural Properties of Composites

#### 3.1.1. Flexural Properties of Composites at Room Temperature

**Figure 6:**
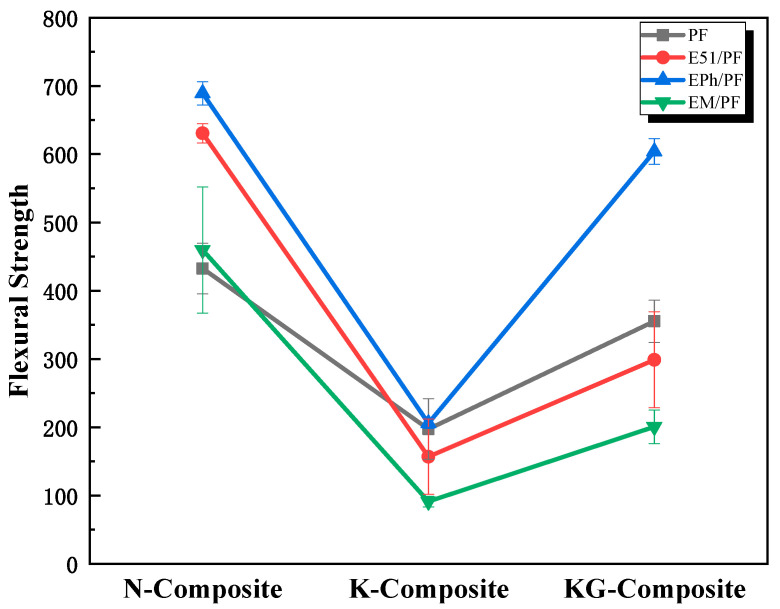
Flexural strength of N-Composite, K-Composite, and KG-Composite systems at room temperature.

Due to the fire resistance of kaolin [32] and graphite at high temperature, the inclusion of these two types of fillers can enhance the resin char yield and reduce fusion between fibers. Therefore, kaolin and kaolin/graphite were chosen for the study. As depicted in Figure 6, 12 types of composites were subjected to testing for the room-temperature flexural properties, utilizing 4 distinct resin matrices and 2 kinds of fillers.

Compared with the K-Composite system and the KG-Composite system, the N-Composite system exhibited higher flexural strength. Furthermore, the flexural strength of the KG-Composite system is higher than that of the K-Composite system, as shown in Figure 6. Kaolin weakens the extent of resin curing, and this weakening effect is mitigated through the reduction in kaolin content and the addition of graphite.

In the N-Composite system, the flexural strength of composites decreased sequentially in EPh/PF/GF, E51/PF/GF, EM/PF/GF, and PF/GF. Based on the early-stage research findings published by the authors of [34,36], it has been concluded that epoxy modified with polydimethylsiloxane exhibited inferior mechanical performance, and resin can be toughened by epoxy modified with polyphenylpropylsiloxane. As a result, the incorporation of Z-6018 enhances the flexural strength of composites compared to E51/PF/GF, whereas the inclusion of SH-203-F notably diminishes the flexural strength of composites compared with E51/PF/GF. In addition, the low strength of PF/GF is attributed to the inherent brittleness of phenolic resin.

In the K-Composite and the KG-Composite systems, EPh improved the room-temperature flexural strength of the phenolic composites, while E51 and EM decreased that of the phenolic composites. The key factor may be the excellent toughening effects of polyphenylpropylsiloxane-modified epoxy and the poor compatibility between kaolin and E51, as well as EM.

#### 3.1.2. Post-Heat Flexural Properties of Composites

**Figure 7:**
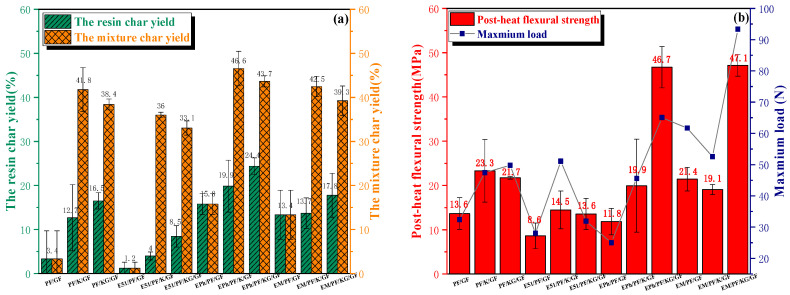
The resin char yield and mixture char yield (**a**); flexural strength and maximum load (**b**) of composites after heat.

The resin char yield, mixture char yield, post-heat flexural strength, maximum load, and bh^2^ (product of width and thickness squared) of 12 composites, respectively, were tested and recorded, as indicated in Table 2 and Figure 7.

A resin matrix with high thermal stability and char yield is necessary but not sufficient for elevating the post-heat flexural strength of composites. The data from Figure 7 demonstrates that the resin char yields of EPh/PF/KG/GF and EM/PF/KG/GF are related to their post-heat flexural strengths. Nonetheless, PF/KG/GF and EPh/PF/K/GF show high resin char yield, while retaining a comparatively low post-heat flexural strength (about 20 MPa).

As outlined in Section 2.4.2, both graphite and resin possess pyrolysis properties, causing different trends in the resin char yield and mixture char yield. Under identical resin systems, both kaolin and graphite exhibited the capacity to mitigate the oxidative pyrolysis of resin. Because of the oxygen consumption of graphite, its superior carbon-retention ability resulted in a sequential increase in the resin char yield of the N-Composite, K-Composite, and KG-Composite systems. The mixture char yield displayed a trend of initial increase followed by decrease for the N-Composite, K-Composite and KG-Composite systems, caused by the pyrolysis of graphite. In addition, the variation tide of the post-heat flexural strength of the PF-Composite and the E51/PF-Composite corresponds to the trend of the mixture char yield of these samples. Although the inclusion of graphite into the EPh/PF-Composite and EM/PF-Composite leads to a slight reduction in the mixture char yield, it contributes to a more complete sample structure with fewer defects and increased dimensional stability after heat, ultimately resulting in the growth of strength. The details about complete sample structure and dimensional stability after heat are presented below. These experimental outcomes imply that kaolin enhances the post-heat flexural strength of composites, while graphite exerts diverse effects on the composites, which depend on the resin systems utilized. To summarize, a resin matrix with great thermal stability and superb char yield is undeniably important, but the deformation caused by the internal pressure of the pyrolysis gas products and the structure after heat are equally critical for determining the post-heat mechanical property.

The post-heat strength of EPh/PF/GF (25N), EPh/PF/K/GF (46N) and EPh/PF/KG/GF (65N) rose continuously; this was mainly attributed to a notable rise in the maximum load, which directly influenced this outcome. Additionally, the greatest dimensional stability of EPh/PF/KG/GF after heat caused the lowest bh^2^ and the highest strength. On the one hand, the smaller maximum load of EPh/PF/GF may come from its low silicon content as well as the crispness and low strength of the generated SiO_2_. On the other hand, in the K-Composite and KG-Composite systems, the presence of kaolin enhanced the post-heat strength in comparison with the N-Composite by impeding fiber fusion and promoting carbon retention at high temperature. In addition, a further increase in the post-heat strength of KG-Composite may be linked to the fact that graphite has better compatibility with the C/Si/O hybrid pyrolysis products [33,35] of siloxane-modified resin than kaolin.

The dimension and maximum load have a simultaneous impact on EM/PF/GF (61N) and EM/PF/K/GF (42N), with the former exhibiting slightly higher post-heat strength than the latter. The high Si content of EM/PF/GF probably leads to this effect. Despite the expansion of EM/PF/GF due to the internal air pressure, the incremental char increases the maximum load, making for slightly higher post-heat strength than that of EM/PF/K/GF. This phenomenon has also been evidenced in the cross-sectional macro-morphology of EM/PF/GF. EM/PF/KG/GF (93N) presented with the highest strength, and the facts that its maximum load is higher than that of others and its bh^2^ is smaller than that of others are responsible for its post-heat strength being nearly equal to that of EPh/PF/KG/GF.

The analysis above indicates that there are two factors that cause direct differences in the post-heat strength. Firstly, the effect of dimension on the post-heat strength after heat is considered. The maximum loads of EPh/PF/KG/GF (65N), EM/PF/GF (61N), PF/KG/GF (50N), and E51/PF/K/GF (51N) are approximately equivalent. However, there was a notable disparity in dimension, as indicated by bh^2^ in Table 2, inducing variations in the post-heat flexural performance of composites. It is evident that dimension stability greatly influences flexural strength. Secondly, the effect of the maximum load on the post-heat strength is also significant. Similar bh^2^ values were observed between PF/K/GF and EPh/PF/GF, and between PF/KG/GF and E51/PF/KG/GF, as well as between E51/PF/KG/GF and EPh/PF/K/GF, while these samples display obvious changes in the maximum load and post-heat strength.

Moreover, according to the flexural strength residual rate of Table 3, the value of E51/PF/KG/GF is 4.8%; this is lower than those of PF/KG/GF (5.8%) and EPh/PF/KG/GF (7.7%). The addition of E51 reduced the heat resistance of the phenolic resin. However, the flexural strength residual rate improved after the epoxy grafting with organosiloxane as a result of the generation of inorganic C/Si/O hybrid pyrolysis products, which slow down the loss of carbon. The flexural strength residual rate of EM/PF/KG/GF was an impressive 23.5%, which is about three times higher than those of the top three. The main factor behind this is the inferior flexural strength of EM/PF/KG/GF at room temperature.

### 3.2. Macroscopic Morphology of Composites after Heat

#### 3.2.1. Macroscopic Morphology of Upper Surface of Composites after Heat

**Figure 8:**
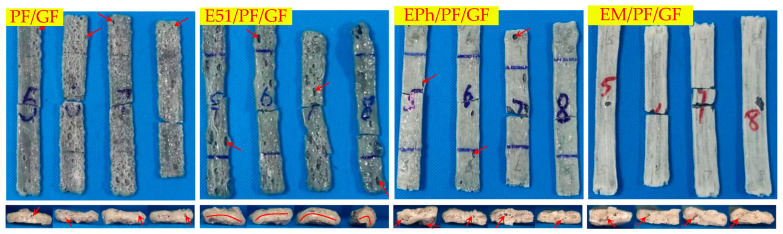
Upper surface and side morphologies of PF/GF, E51/PF/GF, EPh/PF/GF, and EM/PF/GF after heat (the solid arrow points to the pores and volcanic pores, while the solid line indicates distortion).

**Figure 9:**
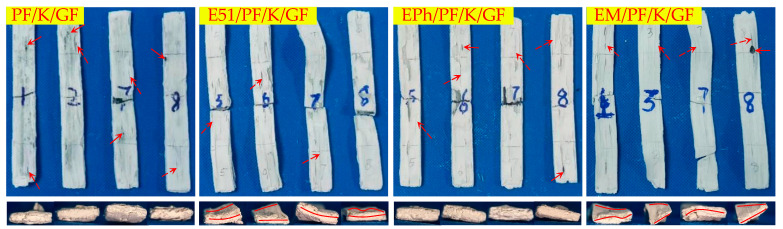
Upper surface and side morphologies of PF/K/GF, E51/PF/K/GF, EPh/PF/K/GF, and EM/PF/K/GF after heat (the solid arrow points to the pores, the dashed arrow points to the linear cracks, and the solid line indicates distortion).

**Figure 10:**
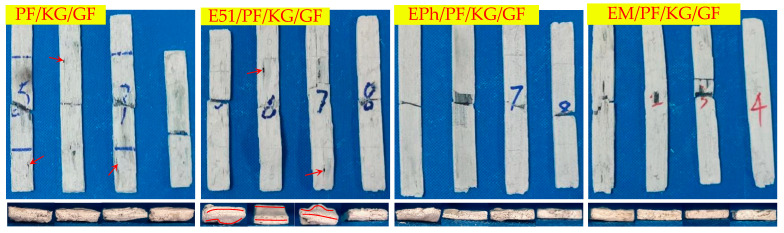
Upper surface and side morphologies of PF/KG/GF, E51/PF/KG/GF, EPh/PF/KG/GF, and EM/PF/KG/GF after heat (the solid arrow points to pores, while the solid line indicates distortion).

Figure 8, Figure 9 and Figure 10 illustrate the macroscopic morphologies of the upper surfaces of the composites after heat. Six distinct phenomena were observed: expansion and contraction, changes in color, the formation of raised edge pores (labelled as volcanic pores below), the formation of non-raised pores (labelled as pores below), distortion, and the occurrence of linear cracks. The release of small molecules from resin pyrolysis accounts for the presence of volcanic pores breaking through the quasi-molten surface and producing pores with dimensions of 2–5 mm; these were primarily found on the upper surface. The pores predominantly manifested on the side, each with a diameter of less than 1 mm. The distortion refers to the deformation of composites. Linear cracks were only present on the upper surface.

It is evident that PF/GF and E51/PF/GF exhibited contraction, with a grayish black as well as glassy surface and numerous volcanic pores distributed; E51/PF/GF underwent significant distortion, with pores on the side; EPh/PF/GF and EM/PF/GF expanded and displayed a smooth, gray-white, glassy surface, with scarcely any volcanic pores, and smaller pores on the side. Because of the presence of E51, the fire resistance of the samples was compromised, ultimately giving rise to the distortion. The samples with siloxane-modified epoxy underwent pyrolysis to form C/Si/O hybrid structures, preventing gas from escaping and causing its gas to release from the side. As a result, this process led to the absence of volcanic pores on the upper surface of samples, with pores forming on the side.

Kaolin exhibits specific sintering properties that obstruct gas emission and the sintering of glass fibers, making the material expand and reduce in contraction; it also lends a loose and white surface, displaying many linear cracks and powder shedding. Despite the presence of several disadvantages, it notably diminishes the occurrence of volcanic pores on the upper surface. The upper surfaces of PF/K/GF and EPh/PF/K/GF illustrated relatively fewer defects than other composites containing kaolin, such as volcanic pores and linear cracks; E51/PF/K/GF and EM/PF/K/GF exhibited numerous linear cracks on the upper surfaces and dramatic distortion. The variation in macroscopic morphology between EM/PF/K/GF and EPh/PF/K/GF may stem from the distinct structures of their C/Si/O hybrid pyrolysis products.

Graphite lacks sintering characteristics, yet it possesses fire-resistance properties and exerts an oxygen-consuming effect. The macroscopic morphologies of the samples after heat were markedly influenced by the introduction of graphite, providing a dense surface without powder shedding. The linear cracks of PF/KG/GF and E51/PF/KG/GF were thus diminished, and the distortion of the latter was comparatively lessened. In addition, the defects of EPh/PF/KG/GF and EM/PF/KG/GF on the upper surface and the side vanished, and their dimensional stability significantly improved. This improvement can be attributed to the oxygen consumption of graphite, which protected the char and thereby achieved structural fire resistance.

#### 3.2.2. Macroscopic Morphology of Cross-Section of Composites after Heat

**Figure 11:**
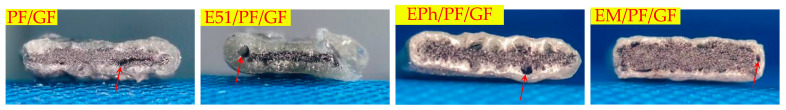
Macroscopic cross-section of PF/GF, E51/PF/GF, EPh/PF/GF, and EM/PF/GF after heat. (The solid arrow points to the vent hole).

**Figure 12:**
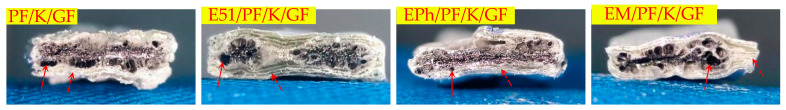
Macroscopic cross-section of PF/K/GF, E51/PF/K/GF, EPh/PF/K/GF, and EM/PF/K/GF after heat. (The solid arrow points to the vent hole, while the dashed arrow points to the layered composite structure).

**Figure 13:**
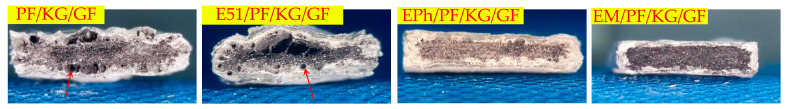
Macroscopic cross-section of PF/KG/GF, E51/PF/KG/GF, EPh/PF/KG/GF, and EM/PF/KG/GF after heat. (The solid arrow points to the vent hole).

Figure 11, Figure 12 and Figure 13 display images of the composites’ cross-sections, comprising the outer layer and inner layer as well as interface, after heat. Clear interfaces appear within all composites. The outer layer is composed of fused glass fibers and other residual substances, while the inner layer consists of black residues.

The outer layers of PF/GF and E51/PF/GF display a gray-black glass state, with a decreased vent pores at the interface, and the pyrolysis gas products release from the upper surface; instead, the outer layers of EPh/PF/GF and EM/PF/GF exhibit a gray-white glass state, featuring numerous vent pores at the interface through which the pyrolysis gas products are discharged.

The outer layers of the K-Composite include a layered composite structure, with vent pores present in both the inner and outer layers as well as at the interface. Moreover, fibers within the center section of E51/PF/K/GF underwent significant fusion and merged into a state. The layered composite structure was produced due to the ability of kaolin to hinder the interlayer sintering of fibers and the release of gas. This facilitates greater porosity and significant deformation in the cross-sections of samples.

When compared with the K-Composite, the non-sintering properties of graphite aid in the gas release and the reduced porosity at the inner layer and interface of the KG-Composite. The interface of PF/KG/GF and E51/PF/KG/GF exhibits many large vent pores compared to that of EPh/PF/KG/GF and EM/PF/KG/GF, which displays no significant defects. This allows for the escape of internal small molecule gases through micro- and nanoscale vent pores.

According to the analysis provided, the composites can be classified according to their macroscopic morphology characteristics, as outlined in Table 4. The summary is as follows:

(1) There are a large number of volcanic pores for PF/GF and E51/PF/GF, presenting on the upper surfaces.
(2) Distortion is observed in the specimens with E51.
(3) When considering the K-Composite, it is evident that they exhibit characteristics such as linear cracks on the upper surface, internal and interfacial vent pores, as well as layered composite structures on the outer layer.
(4) The defects of the upper surfaces of the composites with siloxane-modified epoxy are minimal after heat, and the structural integrity is optimal upon the addition of kaolin/graphite.
(5) The addition of inorganic fillers lowers the propensity of fiber fusion.

The combustion of composites encompasses a range of physical and chemical processes, including the individual and mutual fusion of fibers and fillers, the oxidation and pyrolysis of organic resin, the emission of pyrolysis gas products, and the interaction between pyrolysis products of resin and inorganic fillers. Upon examining the morphologies of the pyrolysis products, it becomes apparent that siloxane-modified epoxy can be incorporated as an organic precursor of inorganic elements into phenolic composites. This method enhances the mechanical properties at room temperature, facilitates the generation of uniform “in situ” products, mitigates material pyrolysis, increases the structural integrity of material, improves the surface morphology of resin-based composites after heat, preserves carbon residue, and inhibits the formation of vent pores at the interface. Inorganic fillers are predominantly employed for the purpose of preventing fiber fusion, and fillers with diverse sintering characteristics have different advantages.

### 3.3. Microscopic Morphologies of Composites after Heat

#### 3.3.1. Microscopic Morphologies of Composite Surfaces after Heat

**Figure 14:**
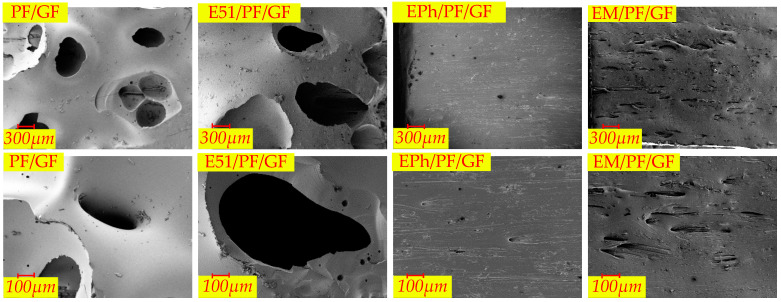
The microscopic morphologies of surfaces of PF/GF, E51/PF/GF, EPh/PF/GF, and EM/PF/GF after heat.

**Figure 15:**
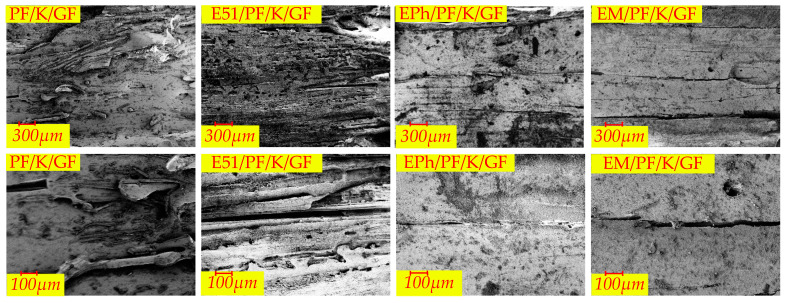
The microscopic morphologies of surfaces of PF/K/GF, E51/PF/K/GF, EPh/PF/K/GF, and EM/PF/K/GF after heat.

**Figure 16:**
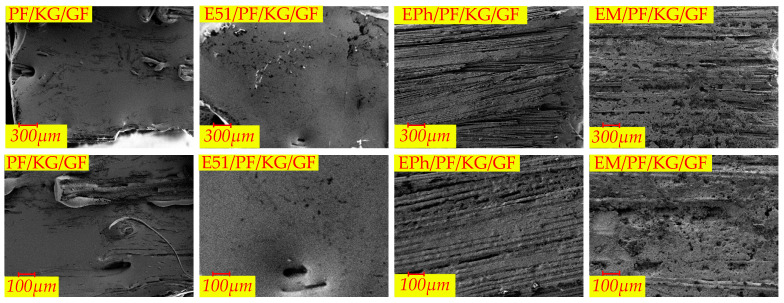
The microscopic morphologies of surfaces of PF/KG/GF, E51/PF/KG/GF, EPh/PF/KG/GF, and EM/PF/KG/GF after heat.

The microscopic morphologies of the surfaces of the composites at 30 and 100 times magnification after heat are depicted in Figure 14, Figure 15 and Figure 16, representing N-Composite, K-Composite, and KG-Composite, respectively.

On the surfaces of PF/GF and E51/PF/GF, numerous large pores (volcanic pores) are observed, while EPh/PF/GF and EM/PF/GF show a few arc-shaped oblique perforations. As stated in Section 2.2.1, the pores serve as pathways for the release of pyrolysis gas products, suggesting that the exhaust channels of the surfaces of samples with the siloxane-modified epoxy are blocked.

PF/K/GF and E51/PF/K/GF display uneven surfaces with cracks and pores, whereas EPh/PF/K/GF and EM/PF/K/GF present relatively flat surfaces with minimal cracks.

Both the surface of PF/KG/GF and the surface of E51/PF/KG/GF are relatively smooth and flat, displaying a sintered state with minimal pores and protrusions. Unlike the former two, the surfaces of EPh/PF/KG/GF and EM/PF/KG/GF present a striped fiber morphology, caused by the incomplete fusion of the superficial fibers. This phenomenon may be attributed to the fact that the siloxane and resin residues together form a “bridge” among the fibers during combustion. The “bridge” isolates external heat and oxygen, leading to improved resin char yield, dimensional stability, and structure density. Consequently, this process significantly enhances the maximum load and post-heat flexural strength of the latter two after heat.

#### 3.3.2. Microscopic Morphology of Cross-Section of Composites after Heat

**Figure 17:**
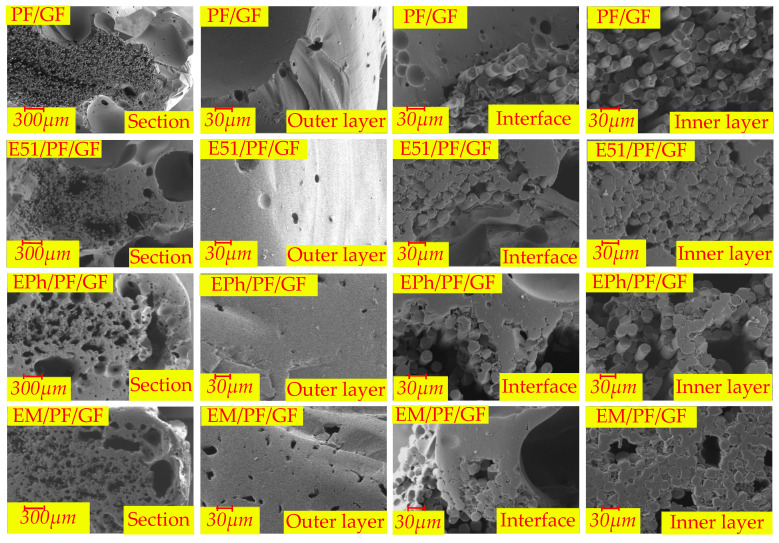
The microscopic morphologies of cross-sections of PF/GF, E51/PF/GF, EPh/PF/GF, and EM/PF/GF after heat.

**Figure 18:**
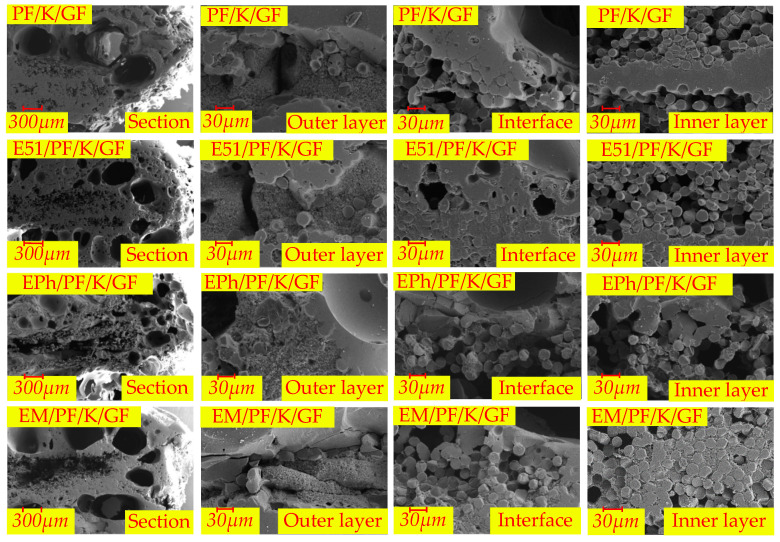
The microscopic morphologies of cross-sections of PF/K/GF, E51/PF/K/GF, EPh/PF/K/GF, and EM/PF/K/GF after heat.

**Figure 19:**
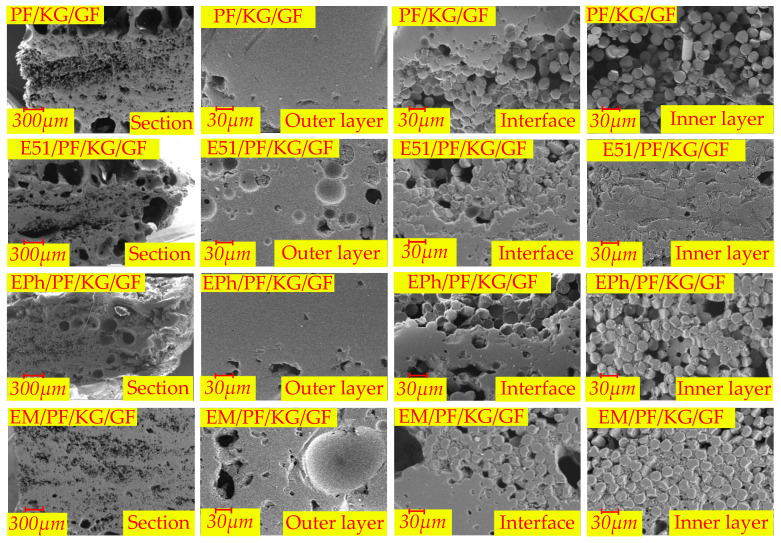
The microscopic morphologies of cross-sections of PF/KG/GF, E51/PF/KG/GF, EPh/PF/KG/GF, and EM/PF/KG/GF after heat.

The microscopic morphologies of the cross-sections of the N-Composite, K-Composite, and KG-Composite after heat are presented in Figure 17, Figure 18 and Figure 19, respectively. These figures provide detailed views of the cross-section, inner layer, interface, and outer layer.

In Figure 17, it is apparent that the outer layers of PF/GF and E51/PF/GF feature numerous volcanic pores protruding from surface, and the interface of these composites is relatively continuous; the fibers of the inner layer contain gaps and vent pores. In contrast, the dense outer layer is evident in both EPh/PF/GF and EM/PF/GF, with numerous vent pores observed at the interface and in the inner layer. The changes in microstructure are related to the alteration trend of post-heat flexural strength.

In Figure 18, kaolin, which is not able to sinter with fibers, and the pyrolysis products of resin cause a layered composite structure of glass–filler–glass in the outer layer of the K-Composite. This explains the development of local layered composite structures on a macroscale. In addition, a considerable number of large vent pores emerge at the interface of the K-Composite.

Figure 19 demonstrates the presence of large vent pores at the interface of PF/KG/GF and E51/PF/KG/GF. On the contrary, EPh/PF/KG/GF and EM/PF/KG/GF acquire a relatively dense material structure after heat, with fewer and smaller vent pores. The phenomenon may be a consequence of the favorable compatibility between graphite and C/Si/O hybrid pyrolysis products of the siloxane-modified resin and their distinct sintering effects. The sintered substance possesses a certain load-carrying ability, and the pressure generated by the pyrolysis gas products is inadequate, making the vent pores expansive, thus leading to the formation of numerous microscale vent pores.

Upon examining the 12 materials, it is clear that the extent of fusion of the inner glass fibers is smaller than that of the outer layer. There is a positive correlation between the depth from the surface to the interior and the extent of fusion of glass fibers. The closer the resin is to the outer side, the higher the extent of resin pyrolysis is; consequently, there is a greater likelihood of fusion glass fiber occurring. Furthermore, only the inner glass fibers of EPh/PF/KG/GF and EM/PF/KG/GF exhibit no substantial fusion. The tight sintering of the outer layers of the two materials increases structural density and reduces the intrusion of oxygen and heat, thereby contributing to the highest post-heat flexural strength.

According to the analysis of microscopic morphology following pyrolysis, it is apparent that the in situ oxidation–pyrolysis products of siloxane-modified epoxy and kaolin both reduce the formation of volcanic pores and the pyrolysis of carbon residue and the tendency of fibers fusion; they also improve the surface morphology of polymer composites after heat and enhance their structural integrity, ultimately leading to an increase in post-heat flexural strength. Additionally, the inclusion of graphite contributes to improvements in the surface densification, structural integrity, and post-heat flexural strength. Lastly, the co-sintering effect between the C/Si/O hybrid pyrolysis products brought by siloxane-modified epoxy and graphite results in a notable increase in the post-heat flexural strength of the silicon-modified resin system.

### 3.4. The Pyrolysis Process and Residual Structure Model of Composites

During the heat treatment, the underlying physical and chemical processes of composites can be deduced from the macro/micromorphology of the surfaces, as well as from the macro/micro cross-sections, as demonstrated below.

(1) When exposed to high temperatures, the resins undergo oxidation and pyrolysis, and subsequently the pyrolysis gas releases through the gaps between the fibers.
(2) Over time, the fusion of fibers occurs, which obstructs the outflow of gases. As it penetrates the molten layer formed by fibers fusion, volcanic pores are generated.
(3) The samples, when incorporating siloxane-modified epoxy, yield C/Si/O pyrolysis products at high temperature, resulting in a slow pyrolysis process that obstructs both gas release and fibers fusion. Similarly, the inclusion of inorganic fillers into the composites has greater difficulties in gas escape and impedes fibers fusion. Therefore, these two types of composites do not generate volcanic pores. But they operate through different mechanisms. In the former case, the C/Si/O pyrolysis products are generated in situ and subsequently interconnect to form homogeneous materials, while in the latter case, the uneven distribution of inorganic fillers brings a limited number of pores.
(4) Sintered fillers, such as kaolin, obstruct fibers fusion and induce sintering shrinkage of materials, causing linear cracks on the surface.
(5) Non-sintered fillers, such as graphite, retard sintering shrinkage of materials, prevent linear cracks on the surface, and consume oxygen to preserve the char.
(6) Graphite also has strong compatibility with the C/Si/O hybrid pyrolysis products of siloxane-modified epoxy. This compatibility mitigates the number of large vent pores at the interface and in the inner layer.

**Figure 20:**
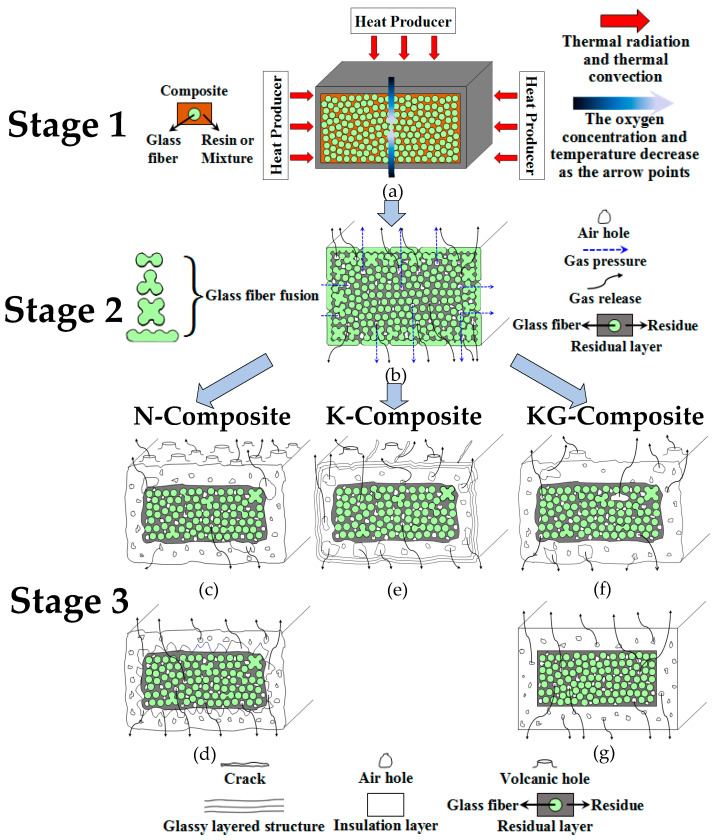
Thermal decomposition process and residual structure model of composites. (**a**–**g**) show the first stage, second stage, and third stage of thermal decomposition process of composites, respectively. Additionally, (**c**–**g**) depict the residual structure of N-Composite, K-Composite and KG-Composite, respectively.

Considering the analysis above, the pyrolysis process and residual structure model of composites depicted in Figure 20 can be ascertained. This model, to some extent, reveals the impacts of resin, kaolin, and kaolin/graphite on the evolution of the morphology, the structural formation, and the mechanical properties of composites after heat.

#### 3.4.1. Three-Stage Pyrolysis Process of Composites

The pyrolysis process can be divided into three stages, each with their own characteristics, as depicted in Figure 20 and in the explanation below.

In the initial phase, as described in Figure 20a, the superficial resin of the samples undergoes pyrolysis, transforming the resin into char due to thermal convection and radiation. The level of pyrolysis in the inner layer is comparatively minimal, with a gradual reduction in oxygen concentration from the exterior to the interior. Moreover, glass fibers are evenly spread throughout the matrix, remaining unfused and in a separated state from each other.

**Figure 21:**
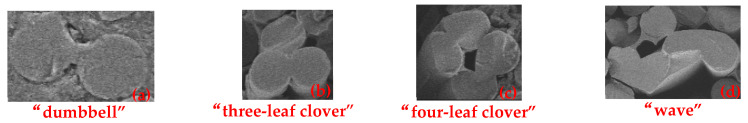
The fusion morphologies of glass fibers of composites during the pyrolysis process. (**a**–**d**) show the fusion morphologies of glass fibers of composites during the pyrolysis process. Additionally, the fusion morphologies of (**a**–**d**) are labeled as “dumbbell” shape, “three-leaf clover” shape, “four-leaf clover” shape and “wave” shape, respectively.

During the second stage, as illustrated in Figure 20b, the absence of carbon substances between the superficial glass fibers of the samples makes these fibers gradually fuse due to the char oxidation. As a consequence, the closer the structure is to the inner layer, the higher the char content and the lower the degree of fiber fusion. On the one hand, the outermost fibers are largely fused together into a “wave” shape. On the other hand, the inner fibers are mostly unaffected, with only a small portion of fibers near the outer side being fused together to form a “dumbbell” shape, a “three-leaf clover” shape and a “four-leaf clover” shape, respectively. These shapes (the fusion morphology of glass fibers) can be also found in the microscopic morphologies described in Section 3.3.2, as displayed in Figure 21a–d.

In Figure 20c–g, the third stage is depicted. After prolonged exposure to high temperatures, the majority of the residual carbon on the surface undergoes oxidation, causing the glass fibers to merge into a continuous glassy material. This material is separated from the internal black residues, forming two phases. Also, it is worth noting that various samples present five residual structures and methods of gas release at this stage.

#### 3.4.2. Residual Structure Model of Composites after Heat

In the case of the N-Composite, the macroscopic surface morphologies of PF/GF and E51/PF/GF show numerous volcanic pores, as depicted in Figure 20c. EPh/PF/GF and EM/PF/GF display a uniform surface morphology, with macroscopic pores forming at the interface (between the inner and outer layers of the cross-section), as illustrated in Figure 20d. The reasons for this are listed below. As the pyrolysis gas breaks through the molten layer, it creates volcanic pores due to the fact that the viscosity of the molten material on the surface of the former two is relatively low. During the pyrolysis process of the latter two, hybrid C/Si/O products are formed, creating a uniform barrier layer exhibiting significant high-temperature rigidity on the surface. This layer effectively prevents the escape of gas and the loss of carbon elements, enabling the converging gas to discharge from the interface and pores to subsequently form at the interface, bringing about the expansion of materials. Accordingly, the resin residue rate and post-heat flexural strength of the former two are lower than these of EM/PF/GF. The inferior post-heat flexural strength of EPh/PF/GF may be attributed to the fact that the formed SiO_2_ is a low-strength and brittle material.

As for the K-Composite (Figure 20e), linear cracks emerge on the surface and a glassy layered structure forms in its outer layer, while the interface between the inner and outer layers has numerous vent pores. The presence of kaolin impedes gas flow and fiber fusion, leading to the accumulation of gas at the interface. This interface is highly sensitive to air pressure, easily resulting in the deformation and formation of pore defects. However, at elevated temperatures, kaolin can also hinder the sintering of glass fibers and preserve the char, thereby enhancing the post-heat flexural strength of the K-Composites compared to the N-Composites, with the exception of EM/PF/K/GF. Notably, the post-heat flexural strength of EM/PF/GF and EM/PF/K/GF is comparable, probably resulting from a higher siloxane content in the former.

Unlike the previous two composite systems, when it comes to the KG-Composite, PF/GF and E51/PF/GF display few macroscopic defects on the surface; yet, large vent pores are still present in the cross-section (Figure 20f). In contrast, EPh/PF/KG/GF and EM/PF/KG/GF exhibit uniform surface morphology without obvious defects. Additionally, the residues in the cross-section are exceptionally dense, and the interface between the inner and outer layers also shows tight bonding (Figure 20g). The oxygen-consuming effect of graphite can protect the char, leading to fewer surface defects and a denser residual structure. Consequently, the post-heat flexural strength of the KG-Composite surpasses that of the N-Composite. But the effect of graphite on the composites depends on whether it contains siloxane-modified epoxy. At high temperatures, the interaction between siloxane-modified epoxy and graphite leads to the development of the densest C/Si/O hybrid pyrolysis structure. This structure tightly envelops the inner substance, safeguarding the char from resin pyrolysis. As a result, the structural integrity and post-heat flexural strength are optimized, thereby achieving the structural fire resistance of composites.

## 4. Conclusions

(1) Initially, the introduction of epoxy into the phenolic composites resulted in an increase in room-temperature strength, a decrease in the post-heat flexural strength, and the appearance of abundant volcanic pores on the surface, along with the distortion of structure. The second thing is that the addition of phenylpropylsiloxane-modified epoxy leads to an increase in room-temperature strength, a decrease in the post-heat flexural strength, the absence of surface defects, and the presence of numerous vent pores in the cross-section. Lastly, the incorporation of the dimethylsiloxane-modified epoxy brings about a slight increase in room-temperature strength, an increase in post-heat flexural strength, the absence of surface defects, and an increase in cross-sectional porosity. The chain flexibility of siloxane-modified epoxy improves the brittleness of phenolic composites at room temperature. Furthermore, at elevated temperatures, the C/Si/O hybrid pyrolysis products impede gas escape, forming a uniform surface without defects. The diminished post-heat flexural strength observed in samples with epoxy modified by phenylpropylsiloxane may be attributed to the fragility and low strength of the generated SiO_2_.
(2) The addition of kaolin into phenolic composites led to a reduction in room-temperature strength, an increase in post-heat flexural strength, the formation of a loose and powdery surface, the emergence of layered composite structures, and large vent pores in the cross-section, as well as the distortion of overall structure; meanwhile, the number of volcanic pores on the surface declined. Additionally, after adding kaolin/graphite, the phenolic composites exhibited reduced strength loss at room temperature and improved post-heat flexural strength, dense surface with minimal powder peeling, and no discernible defects. Although vent pores are present in its cross-section, it was denser than the K-Composite. These phenomena are a result of the fact that kaolin decreases resin curing, while the reduction in kaolin mass improves this weakening effect at room temperature. At high temperatures, kaolin not only hinders the gas escape and sintering of glass fibers, but also retains the char. However, this property of preventing sintering causes material deformation. Moreover, graphite serves to consume oxygen and protect the char.
(3) There is an interactive effect between resin and inorganic fillers on the post-heat flexural strength. The in situ C/Si/O hybrid pyrolysis products of epoxy modified with siloxane and kaolin both blocked gas releasing and glass fiber sintering, and promoted carbon retention, as well as enhancing the post-heat flexural strength of the samples. Due to the poor compatibility between these two substances, after heat the structure underwent the occurrence of distortion and defects. In contrast, the great compatibility and even certain interactions between graphite and C/Si/O hybrid pyrolysis products occurred, causing the formation of micro/nanoscale vent pores and the densification of the material after heat.
(4) The pyrolysis process of phenolic composites reinforced by glass fiber correlates with the oxidation as well as decomposition of matrix and the fusion among glass fibers. In addition, the residual structure model of composites after heat involves structural characteristics such as surface defects, vent pores at the interface of the cross-section, glass fiber fusion, and material deformation. These findings uncover the influence of variables like resin, kaolin, and graphite on the morphology, structure, and mechanical properties of polymer composites after heat.
(5) Based on the investigation of the morphology, structure, and post-heat flexural strength of four diverse resin systems and two various filler systems, it is observed that siloxane-modified epoxy and inorganic fillers share similar effects on the post-heat flexural strengths and morphologies of composites after heat. These materials can improve surface morphology and prevent the loss of char. Nevertheless, the former is more effective, yielding uniformly distributed “in situ” C/Si/O hybrid pyrolysis products, which exhibits the interactive effects with fillers at high temperature. Hence, in order to adapt to the pyrolysis characteristics of siloxane-modified epoxy, optimizing the content and altering the type of inorganic fillers are crucial for the structural fire retardancy of materials. This facilitates the research of the pyrolysis mechanism and the high-temperature utilization of phenolic composites.

## Figures and Tables

**Table 1:** The abbreviation of glass fiber composites and used mass of components.

Composite System	PF-Composite	E51/PF-Composite	EPh/PF-Composite	EM/PF-Composite
N-Composite (No filler)	PF/GF (60 g/270 g)	E51/PF/GF (18 g/42 g/270 g)	EPh/PF/GF (18 g/42 g/270 g)	EM/PF/GF (18 g/42 g/270 g)
K-Composite (Containing kaolin)	PF/K/GF (60 g/30 g/270 g)	E51/PF/K/GF (18 g/42 g/30 g/270 g)	EPh/PF/K/GF (18 g/42 g/30 g/270 g)	EM/PF/K/GF (18 g/42 g/30 g/270 g)
KG-Composite (Containing kaolin/graphite, 24 g and 6 g, respectively)	PF/KG/GF (60 g/30 g/270 g)	E51/PF/KG/GF (18 g/42 g/30 g/270 g)	EPh/PF/KG/GF (18 g/42 g/30 g/270 g)	EM/PF/KG/GF (18 g/42 g/30 g/270 g)

**Table 2:** The maximum load (ML) and bh^2^ data of composites after heat.

Data Type	PF/GF	PF/K/GF	PF/KG/GF	E51/PF/GF	E51/PF/K/GF	E51/PF/KG/GF	EPh/PF/GF	EPh/PF/K/GF	EPh/PF/KG/GF	EM/PF/GF	EM/PF/K/GF	EM/PF/KG/GF
ML/N	32	47	50	28	51	32	25	46	65	61	42	93
bh^2^/mm^3^	220	189	206	291	321	212	190	212	125	262	235	179

Notes: The maximum load (ML) is the max force applied to the specimen in the three-point flexural performance test; bh^2^ is the product of width and thickness squared, representing the effect of dimensional changes on the flexural strength of the specimens after heat.

**Table 3:** Flexural strength residual rate of PF/KG/GF, E51/PF/KG/GF, EPh/PF/KG/GF, and EM/PF/KG/GF.

Data Type	PF/KG/GF	E51/PF/KG/GF	EPh/PF/KG/GF	EM/PF/KG/GF
Flexural strength before heat (MPa)	355.3	298.9	604	200.7
Flexural strength after heat (MPa)	20.6	13.6	46.7	47.1
Flexural strength residual rate (%)	5.8	4.6	7.7	23.5

**Table 4:** Macroscopic morphology characteristics of composites.

Macroscopic Morphology		Composites
Upper surface	Volcanic pores	PF/GF, E51/PF/GF,
	Linear cracks	PF/K/GF, E51/PF/K/GF
	Both flat and straight	EM/PF/GF, EM/PF/GF, EPh/PF/KG/GF, EM/PF/KG/GF
	Smooth	EPh/PF/GF, EM/PF/GF
Side	Distortion	E51/PF/GF, E51/PF/K/GF, E51/PF/KG/GF, EM/PF/K/GF
	Pores	PF/GF, EPh/PF/GF, EM/PF/GF
Cross-section	Vent pores in the inner layer	PF/K/GF, E51/PF/K/GF, EPh/PF/K/GF, EM/PF/K/GF
	Vent pores at the interface	PF/GF, E51/PF/GF, EPh/PF/GF, EM/PF/GF, PF/K/GF, E51/PF/K/GF, EPh/PF/K/GF, EM/PF/K/GF, PF/KG/GF, E51/PF/KG/GF
	No obvious defects	EPh/PF/KG/GF, EM/PF/KG/GF
	Outer glassy state	PF/GF, E51/PF/GF, EPh/PF/GF, EM/PF/GF
	Outer layered composite structure	PF/K/GF, E51/PF/K/GF, EPh/PF/K/GF, EM/PF/K/GF

## Data Availability

Data is contained within the article.
